# Supplementary material for: Predominance of Recombinant Norovirus Strains in Greece, 2016–2018
Source: Microorganisms. 2023 Nov 29;11(12):2885. doi: 10.3390/microorganisms11122885 (PMC10745620; doi:10.3390/microorganisms11122885)
Supplement: Supplementary file 1 [file microorganisms-11-02885-s001.zip › microorganisms-2615727-supplementary.pdf]

Table 1. Norovirus strains detected in Greece and elsewhere in the world that were used for the phylogenetic comparison shown in the dendrogram of **Figure 3** and their GenBank Accession Nos.

GenBank Accession No.	Isolate	Date of Detection	Genotype ORF2 (VP1)
**KU821032**	32574/ATH/GII.2/2015	Apr-2015	GII.2
**MK073889**	Hu/USA/GII.P4NewOrleans-GII.4Sydney/St.Louis-0113/2016	Apr-2016	GII.4
**KU821033**	99066/ATH/GII.4/Sydney_2012/2013	Jun-2013	GII.4
**KU821034**	100489/ATH/GII.4/Sydney_2012/2015	Jun-2015	GII.4
**KU821043**	857/ATH/GII.2/2014	Oct-2014	GII.2
**KU821044**	871/ATH/GII.2/2014	Oct-2014	GII.2
**KU954110**	Hu/GI.2/Kaohsiung/16-AF-2/TW/2016	Feb-2016	GI.2
**MF615618**	Hu/GI.P2-GI.2/RUS/Novosibirsk/NS16-A819/2016	Dec-2016	GI.2
**MK280881**	Norovirus/Hu/GI.P2_GI.2/NSW1462/AU/2017	Aug-2017	GI.2
**KU821045**	66230/ATH/GI.2/2014	Nov-2014	GI.2
**AB279570**	GII.2/Hu/OCS020289/JP/2002	2002	GII.2
**KU821046**	78313/ATH/GII.2/2014	Oct-2014	GII.2
**KU821047**	86935/ATH/GII.2/2014	Oct-2014	GII.2
**MK764019**	Hu/US/GII.Pe-GII.4Sydney/Arlington0551/2015	Mar-2015	GII.4
**KU821048**	90353/ATH/GII.4/2014	Oct-2014	GII.4
**MN461124**	20171213_AME_05_GII.Pe_GII.4/2017	Dec-2017	GII.4
**KU821049**	94088/ATH/GII.4/2015	Mar-2015	GII.4
**MT505552**	Hu/GII.P7-GII.6/22632/BLR/2020	Feb-2020	GII.6
**KU821050**	97274/ATH/GII.6/2014	Oct-2014	GII.6
**KX950926**	Hu/GII/AUS/GII.P7_GII.6/Queensland322/2013	Dec-2013	GII.6
**KU821051**	98928/ATH/GII.6/2015	Feb-2015	GII.6
**MZ292794**	Hu/US/GII.8[P8]/CA-RGDS-1133/2014	Jul-2014	GII.8
**MN461148**	20180413_DE_03_GII.P8_GII.8/2018	Apr-2018	GII.8
**KU821052**	94775/ATH/GII.8/2015	May-2015	GII.8
**KU821053**	100126/ATH/GII.4/Sydney_2012/2015	May-2015	GII.4
**MN461127**	20171222_MWE_02_GII.Pe_GII.4/2017	Dec-2017	GII.4
**KU821054**	100539/ATH/GII.4/2015	Jun-2015	GII.4
**KP871750**	80775/ATH/GII.3/2013	Dec-2013	GII.3
**OL898510**	Hu/US/GII.4Sydney[P31]/Nashville13089/2015	Feb-2015	GII.4
**KP871754**	91430/ATH/GII.4/Sydney_2012/2013	Aug-2013	GII.4
**MN461095**	20151214_EI_01_GII.Pe_GII.4/2015	Dec-2015	GII.4
**KP871756**	93555/ATH/GII.4/Sydney_2012/2013	Dec-2013	GII.4
**KP871766**	907/ATH/GII.4/2013	Nov-2013	GII.4
**KP871769**	690/ATH/GII.4/2013	Aug-2013	GII.4
**KP871772**	676/ATH/GII.4/2013	Aug-2013	GII.4
**KP871773**	661/ATH/GII.4/2013	Aug-2013	GII.4
**KP871782**	347/ATH/GII.4/2013	May-2013	GII.4
**KP871790**	241/ATH/GII.4/Sydney_2012/2013	Mar-2013	GII.4
**KP871751**	82396/ATH/GII.4/2014	Jan-2014	GII.4
**KP871753**	89556/ATH/GII.4/Sydney_2012/2013	Apr-2013	GII.4
**KP871755**	91866/ATH/GII.4/Sydney_2012/2013	Sep-2013	GII.4
**KR074181**	GII/Hu/BRA/GII.P16-GII.3/RS19355/2011	2011	GII.3
**KP871757**	94462/ATH/GII.3/2014	Feb-2014	GII.3
**KP871758**	96225/ATH/GII.4/2014	Jun-2014	GII.4
**KF730316**	Hu/GII.2/E24/Saitama/1999	Dec-1999	GII.2
**LC048955**	Hu/GII.2/3-185/Tokyo/JPN/1994	1994	GII.2
**MH218657**	GII.2/NORO_196_18_10_2015	Oct-2015	GII.2

**KP871759**	96354/ATH/GII.2/2014	Jun-2014	GII.2
**KJ504417**	GII/Hu/ESP/GII.P16_GII.3/SanSebastian131919/2012	Oct-2012	GII.3
**KP871760**	40176/ATH/GII.3/2013	Dec-2013	GII.3
**KP871761**	70941/ATH/GII.4/2013	Dec-2013	GII.4
**MK764020**	Hu/US/GII.P16-GII.3/Pittsylvania0388/2014	Mar-2014	GII.3
**KP871763**	1069/ATH/GII.3/2013	Dec-2013	GII.3
**KP871764**	709/ATH/GII.4/2013	Aug-2013	GII.4
**KP871765**	769/ATH/GII.4/2013	Sep-2013	GII.4
**KP871767**	908/ATH/GII.4/Sydney_2012/2013	Nov-2013	GII.4
**LC726030**	GII.4Hu/GII.4[P31]/205/Tokyo/Japan/2021	Jun-2021	GII.4
**KP871768**	911/ATH/GII.4/Sydney_2012/2013	Nov-2013	GII.4
**KP871770**	694/ATH/GII.4/2013	Aug-2013	GII.4
**KP871771**	699/ATH/GII.4/2013	Aug-2013	GII.4
**KP871774**	479/ATH/GII.4/Sydney_2012/2013	Jun-2013	GII.4
**KP871775**	483/ATH/GII.4/Sydney_2012/2013	Jun-2013	GII.4
**KP871777**	407/ATH/GII.4/Sydney_2012/2013	May-2013	GII.4
**MK753019**	Hu/US/GII.Pe-GII.4Sydney/HoodRiver0382/2013	Dec-2013	GII.4
**KP871778**	413/ATH/GII.4/Sydney_2012/2013	May-2013	GII.4
**KP871780**	439/ATH/GII.4/2013	Jun-2013	GII.4
**LC620997**	Hu/GII/JPN/GII.4[P31]/SMUL16-26/2016	Apr-2016	GII.4
**KP871781**	450/ATH/GII.4/2013	Jun-2013	GII.4
**KP871784**	369/ATH/GII.4/Sydney_2012/2013	May-2013	GII.4
**KX657733**	C013/GII.Pe/GII.4ORF1/2016	Mar-2016	GII.4
**KP871785**	286/ATH/GII.4/2013	Apr-2013	GII.4
**KP871786**	340/ATH/GII.4/Sydney_2012/2013	Apr-2013	GII.4
**KP871787**	265/ATH/GII.4/2013	Apr-2013	GII.4
**KT224481**	Hu/GII.Pg-GII.1/RUS/Omsk/O1408/2012	Mar-2012	GII.1
**MN461142**	20180223_DE_03_GII.Pg_GII.1/2018	Feb-2018	GII.1
**KP871788**	251/ATH/GII.1/2013	Mar-2013	GII.1
**MN461142**	20180223_DE_03_GII.Pg_GII.1/2018	Feb-2018	GII.1
**KP871788**	251/ATH/GII.1/2013	Mar-2013	GII.1
**MN960210**	17131092/NoV/GII.P31/GII.4Sydney/27-Mar-17/CHN/2017	Mar-2017	GII.4
**KP871789**	236/ATH/GII.4/Sydney_2012/2013	Mar-2013	GII.4
**KP871791**	208/ATH/GII.4/2013	Mar-2013	GII.4
**KP871792**	210/ATH/GII.4/Sydney_2012/2013	Mar-2013	GII.4
**KP871793**	138/ATH/GII.4/2014	Feb-2014	GII.4
**MK614061**	Hu/JSNJOB1013/CHN/2017	Oct-2017	GII.2
**LC386002**	Hu/GII.P7_GII.14/SCGY17-D7/Sichuan/CHN/2017	May-2017	GII.14
**KR904230**	GII/Hu/ZA/GII.P7-GII.14/Empangeni_11798/2013	Jul-2013	GII.14
**MN960218**	17131118/NoV/GII.P16/GII.2/21-Jun-17/CHN/2017	Jun-2017	GII.2
**MH393583**	Hu/BRA/GI.P1-GI.1/RJ_26432/2017	Feb-2017	GI.1
**MK762637**	Hu/US/GII.P16-GII.4/Sydney_2012/2015	Dec-2015	GII.4
**KU821040**	22645/ATH/GII.4/2014	Dec-2014	GII.4
**LC621052**	Hu/GII/JPN/GII.4[P31]/Ni16-23/2016	Jul-2016	GII.4
**MH218629**	NORO_158_01_04_2015/Sydney_2012/2015	Apr-2015	GII.4
**MN308020**	186_GII.Pe_GII.4_Araguaina/Sydney_2012/2014	Mar-2014	GII.4
**MN461118**	20171208_FE_02_GII.Pe_GII.4/Sydney_2012/2017	Dec-2017	GII.4
**MK753007**	Hu/US/GII.P2-GII.2/Washington0526/2016	Jun-2016	GII.2
**MN602961**	GII/Sew/SP/GII.2/R16/2017	Jan-2017	GII.2
**MK886710**	Hu/GII.P16-GII.2/RUS/Novosibirsk/NS18-A1593/2018	Apr-2018	GII.2
**MH279827**	GII.P7_GII.6b/PR3020/ITA/2015	Jul-2015	GII.6
**MK762570**	Hu/US/GII.P16-GII.4/Sydney_2012/CS1010/2017	Mar-2017	GII.4
**MN461153**	20180420_AME_06_GII.P2_GII.2/2018	Apr-2018	GII.2
**MH218671**	NORO_213_16_12_2015	Dec-2015	GII.3

**LC213899**	Hu/GII/JP/GII.P16-GII.2/Ibaraki607/2016	Dec-2016	GII.2
**MK764018**	Hu/US/GII.P16-GII.4/Sydney_2012/Stafford0184/2016	Jan-2016	GII.4
**MK764021**	Hu/US/GII.P16-GII.4/Sydney_2012/Loudoun0411/2017	Jul-2017	GII.4
**LC413800**	Hu/GII/JP/GII.P16_GII.2/OsakaFB516/2017	Mar-2017	GII.2
**MN602963**	GII/Sew/SP/GII.2/R20/2017	Feb-2017	GII.2
**MG893002**	Hu/GII.P16-GII.2/RUS/Novosibirsk/NS17-A1317/2017	Oct-2017	GII.2
**MK886703**	Hu/GII.P16-GII.2/RUS/Novosibirsk/NS18-A1485/2018	Mar-2018	GII.2
**LC621078**	Hu/GII/JPN/GII.4[P31]/Ni17-37/2017	Jul-2017	GII.4
**LC621143**	Hu/GII/JPN/GII.4[P31]/M17-15/2017	Oct-2017	GII.4
**MH271650**	Hu/GII.P7-GII.6/32814.1/GO/BR/2010	Aug-2010	GII.6
**OP518602**	GII/Hu/RU/GII.4Sydney[P16]/Nizhny_Novgorod688/2019	May-2019	GII.4
**MT492043**	Hu/SP/GII.4Sydney[P16]/3994.Valencia/2019	Nov-2019	GII.4
**LC406326**	GIIHu/JP/GII.P7_GII.7/OsakaS08/2016	Jul-2016	GII.7
**MH279829**	GII.P7_GII.7/PA91/ITA/2012	Feb-2012	GII.7
**MT501859**	Hu/ES/GII.7[P7]/4019Valencia/2020	Jan-2020	GII.7
**MH469174**	Hu/Guangzhou/GZ2016-L528/CHN/2016	Aug-2016	GII.4
**OM185508**	RSBS94.18/1894027/2018	Nov-2018	GII.4
**MT126392.1**	83699/ATH/GII.6	Jan-2016	GII.6
**MT126388.1**	109375/ATH/GII.4	Jan-2016	GII.4
**MT126407.1**	107059/ATH/GII.14	Jun-2016	GII.14
**MT126405.1**	58920/ATH/GII.4	Aug-2016	GII.4
**MT126384.2**	90152/ATH/GII.2	Sep-2016	GII.2
**MT126402.1**	108956/ATH/GII.4	Sep-2016	GII.4
**MT126389.1**	98562/ATH/GII.2	Sep-2016	GII.2
**MT126393.1**	108094/ATH/GII.2	Oct-2016	GII.2
**MT126398.1**	100472/ATH/GII.4	Oct-2016	GII.4
**MT129796.1**	84504/ATH/GI.1	Oct-2016	GI.1
**MT129795.1**	83985/ATH/GI.1	Oct-2016	GI.1
**MT126395.1**	105732/ATH/GII.2	Nov-2016	GII.2
**MT126397.2**	94110/ATH/GII.4	Nov-2016	GII.4
**MT126396.2**	44230/ATH/GII.2	Nov-2016	GII.2
**MT126408.1**	110087/ATH/GII.2	Nov-2016	GII.2
**MT126401.1**	107383/ATH/GII.4	Nov-2016	GII.4
**MT126391.1**	98508/ATH/GII.4	Nov-2016	GII.4
**MT126385.1**	110538/ATH/GII.3	Nov-2016	GII.3
**MT126379.1**	88494/ATH/GII.2	Nov-2016	GII.2
**MT126400.1**	97036/ATH/GII.4	Dec-2016	GII.4
**MT126399.1**	99993/ATH/GII.4	Dec-2016	GII.4
**MT126404.1**	91556/ATH/GII.4	Dec-2016	GII.4
**MT126394.2**	110936/ATH/GII.2	Dec-2016	GII.2
**MT126378.1**	108445/ATH/GII.2	Dec-2016	GII.2
**MT126377.1**	79859/ATH/GII.2	Dec-2016	GII.2
**MT126406.1**	108023/ATH/GII.2	Dec-2016	GII.2
**MT126386.1**	70425/ATH/GII.2	Dec-2016	GII.2
**MT126382.1**	104442/ATH/GII.4	Jan-2017	GII.4
**MT126381.1**	39835/ATH/GII.2	Jan-2017	GII.2
**MT126380.1**	62032/ATH/GII.2	Jan-2017	GII.2
**MT126383.1**	308620/ATH/GII.4	Jun-2017	GII.4
**MT126403.1**	324123/ATH/GII.4	Jul-2017	GII.4
**OP557585.1**	11726/ATH/GII.4/2018	Feb-2018	GII.4
**OP557589.1**	309673/ATH/GII.4/2018	Apr-2018	GII.4
**OP557580.1**	311236/ATH/GII.4/Sydney_2012/2018	Apr-2018	GII.4
**OP557587.1**	309269/ATH/GII.4/Sydney_2012/2018	Apr-2018	GII.4
**OP557584.1**	311053/ATH/GII.4/Sydney_2012/2018	May-2018	GII.4

**OP557579.1**	315727/ATH/GII.7/2018	May-2018	GII.7
**OP557591.1**	335417/ATH/GII.4/Sydney_2012/2018	Jun-2018	GII.4
**OP557590.1**	334868/ATH/GII.4/Sydney_2012/2018	Jun-2018	GII.4
**OP557572.1**	338275/ATH/GII.4/Sydney_2012/2018	Jun-2018	GII.4
**OP557593.1**	341522/ATH/GII.6/2018	Jul-2018	GII.6
**OP557582.1**	340067/ATH.GII.4/Sydney_2012/2018	Jul-2018	GII.4
**OP557575.1**	341414/ATH/GII.4/Sydney_2012/2018	Jul-2018	GII.4
**OP557574.1**	340361/ATH/GII.7/2018	Jul-2018	GII.7
**OP557586.1**	353552/ATH/GII.4/Sydney_2012/2018	Aug-2018	GII.4
**OP557583.1**	345490/ATH/GII.6/2018	Aug-2018	GII.6
**OP557571.1**	346504/ATH/GII.4/2018	Aug-2018	GII.4
**OP557588.1**	368708/ATH/GII.4/Sydney_2012/2018	Sep-2018	GII.4

Table 2. Norovirus strains and GenBank Accession Nos of strains used for the MCC tree construction, model selection and temporal tree construction of GII.4 strains using the Bayesian Markov-Chain Monte Carlo method implemented in the BEAST software, version 2.2.1 and visualized in FigTree v1.4.3.

GenBank Accession No.	Isolate	Year of Detection
**JX023286**	Hu/GII.4/CHDC5191/1974/USA	1974
**FJ537134**	Hu/GII.4/CHDC5191/1974/US	1974
**FJ537135**	Hu/GII.4/CHDC2094/1974/US	1974
**AB684675**	Hu/GII/21-5/Tokyo/1975/JPN	1975
**AB684704**	Hu/GII/52-2/Tokyo/1980/JPN	1980
**AB684705**	Hu/GII/53-1/Tokyo/1980/JPN	1980
**AB684720**	Hu/GII/64-3/Tokyo/1983/JPN	1983
**AB985437**	Hu/GII/2-37/Tokyo/1987/JPN	1987
**EU078406**	Hu/Richmond/1994/USA	1994
**EU078407**	Hu/Houston/1995/USA	1995
**FJ411169**	Hu/GII.4/Wellington/1995/USA	1995
**JQ478407**	Hu/GII.4/1997/USA	1997
**DQ975270**	Hu/GII-4/Osaka/1998/JPN	1998
**AB303923**	Hu/GGII.4/DenHaag015/2000/NL	2000
**AB303924**	Hu/GGII.4/Waddinxveen016/2000/NL	2000
**AB303925**	Hu/GGII.4/Leeuwarden043/2000/NL	2000
**FJ411170**	Hu/GII.4/Henry/2000/USA	2000
**KC462195**	Hu/GII.4/IT00/ITA	2000
**DQ676861**	Hu/GII-4/123027/2001/UK	2001
**AB303927**	Hu/GGII.4/Utrecht058/2001/NL	2001
**AB504306**	Hu/GII.4/Hiroshima/19/2001/JPN	2001
**KJ407076**	Hu/GII.4/HS66/2001/USA	2001
**EU078408**	Hu/Lonaconing/2001/USA	2001
**AB294778**	Hu/GII-4/Matsudo/021071/2002/JP	2002
**AB303929**	Hu/GGII.4/EmmenE006/2002/NL	2002
**EU096506**	Hu/Norovirus/GII-4/Mohacs1147/2002/HUN	2002
**EU096507**	Hu/Norovirus/GII-4/Kiskunhalas1264/2002/HUN	2002
**EU096510**	Hu/Norovirus/GII-4/Cegled1603/2002/HUN	2002
**EU096515**	Hu/Norovirus/GII-4/Tiszatelek982/2002/HUN	2002
**EU310927**	Hu/Houston/TCH186/2002/US	2002
**EU876872**	Hu/GII.4/PA15R-/2002/I	2002
**EU876890**	Hu/GGII.4/Dijon-E1057/2002/FRA	2002
**FJ538900**	Hu/GII.4/Dijon/E872/2002/FRA	2002
**KU182481**	10504/2002/TUN	2002
**KU182482**	11453/2002/TUN	2002
**KU182483**	1307/2002/TUN	2002
**KX722422**	Hu/GII.4/VIG476/2003/BRA	2003
**KX722423**	Hu/GII.4/VIG516/2003	2003
**KX722424**	Hu/GII.4/VIG603/2003/BRA	2003
**KX722426**	Hu/GII.4/PID18000/2004/BRA	2004
**KX722427**	Hu/GII.4/PID18058/2004/BRA	2004
**KX722428**	Hu/GII.4/PID18133/2004/BRA	2004
**KP784691**	GII/Hu/ZAF/2009/GII.P4_GII.4/Johannesburg/4175	2009
**KP871769**	690/ATH/GII.4	2013
**KP871766**	907/ATH/GII.4	2013
**KP871782**	347/ATH/GII.4	2013
**KP871773**	661/ATH/GII.4	2013

**KP871790**	241/ATH/GII.4	2013
**LC177655**	GII/Hu/VNM/2013/GII.Pe_GII.4 Sydney 2012/NVN13.400	2013
**LC177656**	GII/Hu/VNM/2013/GII.Pe_GII.4	2013
**KX354092**	Hu/GII.4_NewOrleans/2013-SP-0461_040313_WI/2013/USA	2013
**KX354093**	Hu/GII.4_NewOrleans/2013-SP-0462_040313_WI/2013/USA	2013
**KX371608**	GII/Hu/US/2013/GII.4_DenHaag/2013-SP-0460_031913_WI	2013
**KX371609**	GII/Hu/US/2013/GII.4_Sydney/2013-SP-0471_091513_CS	2013
**KX371610**	GII/Hu/US/2013/GII.4_Sydney/2013-SP-0479_091613_CS	2013
**KP871751**	82396/ATH/GII.4	2014
**KU821048**	90353/ATH/GII.4	2014
**KU821042**	420/ATH/GII.4	2014
**KU821041**	313/ATH/GII.4	2014
**KP871793**	138/ATH/GII.4	2014
**KP871758**	96225/ATH/GII.4	2014
**KX372682**	Hu/GII.4/AH14022/2014/CHN	2014
**KX372683**	Hu/GII.4/AH14023/2014/CHN	2014
**KM268078**	Hu/GII/Hong Kong/2014/GII.4/CUHK-NS-226	2014
**KM268079**	Hu/GII/Hong Kong/2014/GII.4/CUHK-NS-229	2014
**KM268080**	Hu/GII/Hong Kong/2014/GII.4/CUHK-NS-235	2014
**KU821049**	94088/ATH/GII.4	2015
**MG786781**	Hu/GII.4/DBM15-156/2015/THA	2015
**KU683739**	Hu/GII.4/Lishui02/2015/CHN	2015
**KY341923**	Hu/GII.4/13164/2015/HUN	2015
**KY407189**	Hu/015I03/ZS/GD/CHN/2016	2016
**MF599260**	groundwater/GII-4/Gangwon/2016/KR	2016
**MF599261**	groundwater/GII-4/Gwangju/2016/KR	2016
**MT126391**	98508/ATH/GII.4	2016
**MT126405**	58920/ATH/GII.4	2016
**MT126404**	91556/ATH/GII.4	2016
**MT126402**	108956/ATH/GII.4	2016
**MT126388**	109375/ATH/GII.4	2016
**MT126383**	308620/ATH/GII.4	2017
**MT126382**	104442/ATH/GII.4	2017
**KY679171**	GII/Hu/HK/2017/GII.Pe-GII.4/CUHK-NS-1303	2017
**KY679172**	GII/Hu/HK/2017/GII.P4-GII.4/CUHK-NS-1310	2017
**KY679173**	GII/Hu/HK/2017/GII.Pe-GII.4/CUHK-NS-1322	2017
**OP557569**	350264/ATH/GII.4/2018	2018
**OP557572**	338275/ATH/GII.4/Sydney_2012/2018	2018
**OP557571**	346504/ATH/GII.4/2018	2018
**OP557575**	341414/ATH/GII.4/Sydney_2012/2018	2018
**OP557578**	372354/ATH/GII.4/2018	2018
**LC521577**	2018/TH/S11_58	2018
**LC521578**	2018/TH/S11_59	2018
**LC521580**	2018/TH/S11_113	2018
**LC521603**	2018/TH/S7_183	2018
**LC573391**	2018/TH_DBM_18_438	2018
**OP557570**	347212/GII.4/Sydney_2012/2018	2018
**LC521591**	2019/TH/S15_82	2019
**LC521592**	2019/TH/S15_86	2019
**LC521597**	2019/TH/S15_52	2019
**MN923215**	ORF2_233/2019	2019
**LC644993**	Hu/GII.4[P31]/1503F/Tokyo/2021/Japan	2021

Temporal signal evaluationby the root-to-tip analysis results, using TempEst program in Beast v1.10.4 package is shown at the following figure:

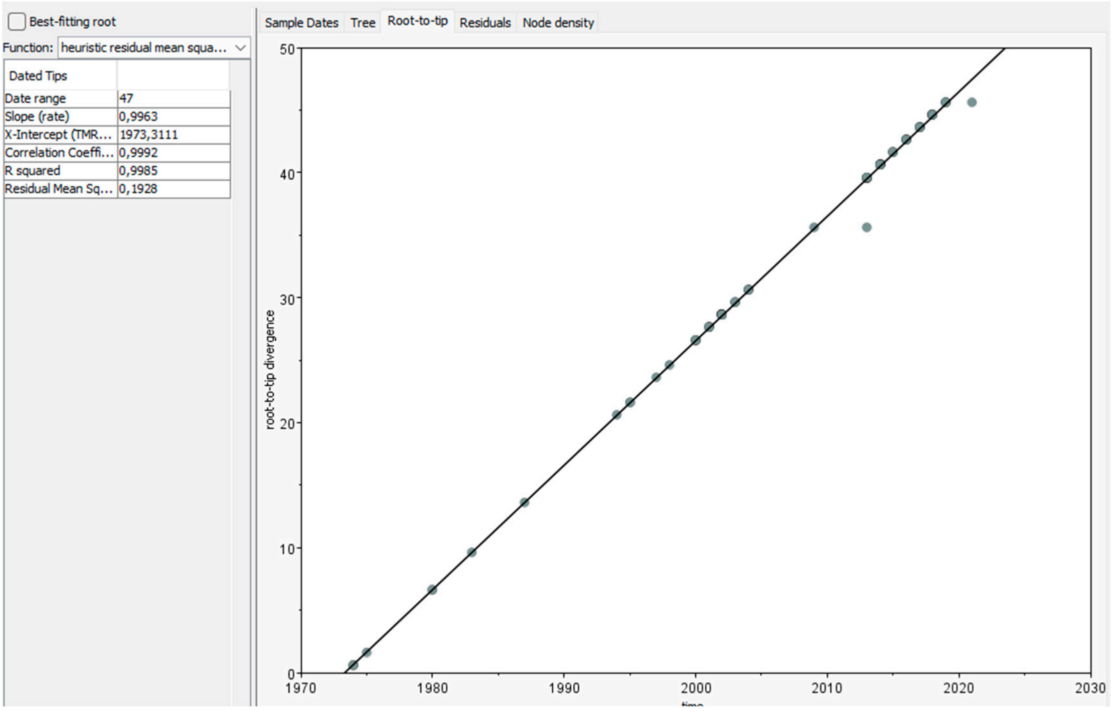

Table 3. Norovirus strains and GenBank Accession Nos of strains used for the MCC tree construction, model selection and temporal tree construction of GII.2 strains using the Bayesian Markov-Chain Monte Carlo method implemented in the BEAST software, version 2.2.1 and visualized in FigTree v1.4.3.

GenBank Accession No.	Isolate	Year of Detection
**MF405169**	Hu/US/1971/GII.Pf__GII.2/HenrytonSP17	1971
**KC597138**	Hu/GII.2/CHDC2596/1975/USA	1975
**KF429769**	Hu/GII.2/SnowMountRS/1975/USA	1975
**JX846925**	Hu/GII.2/KL109/1978/MYS	1978
**KX452702**	Hu/GII.P12/GII.2/24333F17/1983/PA/BRA	1983
**KX722397**	Hu/GII.2/COD401/1991/BRA	1991
**AB281081**	Hu/GII.2/Coevorden191S/1999/NL	1999
**AB281083**	Hu/GII.2/Delft48M/2000/NL	2000
**AB281082**	Hu/GII.2/DenHaag37/2000/NL	2000
**AB281085**	Hu/GII.2/Zwolle25E/2001/NL	2001
**AB281084**	Hu/GII.2/Leeuwarden15/2001/NL	2001
**KC998960**	Hu/GII.2/TCH-560/USA/2002	2002
**AB281087**	Hu/GII.2/Rotterdam39E/2002/NL	2002
**AB281086**	Hu/GII.2/Heerlen7E/2002/NL	2002
**AB281088**	Hu/GII.2/Leeuwarden71/2003/NL	2003
**JN654724**	Hu/GII.2/Vellore/CRI3672/2003	2003
**EU392248**	Hu/GII.2/10488/2004/IRL	2004
**LC209464**	Hu/GII/JP/2004/GII.P2_GII.2/Hokkaido-13	2004
**LC209435**	Hu/GII/JP/2004/GII.P12_GII.2/Tochigi-92	2004
**HM055932**	Hu/GII.2/Hermita/822/2005/NIC	2005
**AB662853**	Hu/GII.2/OC05145/2005/JP	2005
**AB281090**	Hu/GII.2/Vaals87/2005/NL	2005
**LC209462**	Hu/GII/JP/2006/GII.P2_GII.2/Hokkaido-14	2006
**LC016597**	Hu/JP/2006/GII.2/Sakai/SC-3	2006
**HM055937**	Hu/GII.2/rural/824/2006/NIC	2006
**KR074155**	GII/Hu/BRA/2007/GII.P2-GII.2/RS13519	2007
**GU339317**	Hu/GII.2/Cuernavaca/50031/2007/MEX	2007
**AB662869**	Hu/GII.2/OH08029-2/2008/JP	2008
**AB662868**	Hu/GII.2/OH08020/2008/JP	2008
**HQ003269**	Hu/GII.2/Nizhny Novgorod/1414/2008/RUS	2008
**KR074171**	GII/Hu/BRA/2008/GII.P2-GII.2/RS15036	2008
**AB662886**	Hu/GII.2/OH09035/2009/JP	2009
**AB662885**	Hu/GII.2/OH09034/2009/JP	2009
**AB662874**	Hu/GII.2/OC09109-2/2009/JP	2009
**LC209461**	Hu/GII/JP/2009/GII.P16_GII.2/Kanagawa-49	2009
**KJ156608**	GII.2/Nanning147/2010/CHN	2010
**KJ156606**	GII.2/Nanning127/2010/CHN	2010
**AB662902**	Hu/GII.2/OH10031/2010/JP	2010
**AB662895**	Hu/GII.2/OH10015-2/2010/JP	2010
**LC209459**	Hu/GII/JP/2010/GII.P16_GII.2/Kanagawa-51	2010
**KY200631**	Human/MAR/MA029/2011/GII.2	2011
**KX608872**	Hu/GII.2/NIH-P.1/2011/US	2011
**KJ156609**	GII.2/Nanning200/2011/CHN	2011
**LC209479**	Hu/GII/JP/2011/GII.P16_GII.2/Ehime-45	2011
**LC209471**	Hu/GII/JP/2011/GII.P16_GII.2/Hiroshima-26	2011

**LC209468**	Hu/GII/JP/2011/GII.P16_GII.2/Yamaguchi-4	2011
**LC145794**	Hu/GII.2/Hiroshimacity2_2012_JP	2012
**LC145793**	Hu/GII.2/Osakacity5_2012_JP	2012
**LC145792**	Hu/GII.2/Saitama5_2012_JP	2012
**LC209478**	Hu/GII/JP/2012/GII.P16_GII.2/Ehime-46	2012
**LC209466**	Hu/GII/JP/2012/GII.P16_GII.2/Hokkaido-18	2012
**LC209446**	Hu/GII/JP/2012/GII.P16_GII.2/Saitama-121	2012
**LC209477**	Hu/GII/JP/2013/GII.P16_GII.2/Ehime-6	2013
**LC209476**	Hu/GII/JP/2013/GII.P16_GII.2/Ehime-8	2013
**LC209455**	Hu/GII/JP/2013/GII.P16_GII.2/Miyagi-8	2013
**MH218733**	NORO_94_23_10_2014	2014
**LC145803**	Hu/GII.2/Osaka5_2014_JP	2014
**LC145799**	Hu/GII.2/Akita7_2014_JP	2014
**LC209470**	Hu/GII/JP/2014/GII.P16_GII.2/Hiroshima-30	2014
**LC209469**	Hu/GII/JP/2014/GII.P2_GII.2/Yamaguchi-014	2014
**LC209458**	Hu/GII/JP/2014/GII.P16_GII.2/Kanagawa-52	2014
**LC209450**	Hu/GII/JP/2014/GII.P16_GII.2/Osaka-225	2014
**KT962983**	15-DS-4/2015/GII.2	2015
**KU672082**	HU/15S4003/PD/SH/CHN/2015	2015
**LC213885**	Hu/GII/JP/2015/GII.P16-GII.2/Ibaraki197	2015
**LC209457**	Hu/GII/JP/2015/GII.P2_GII.2/Miyagi-63	2015
**MT126377**	79859/ATH/GII.2	2016
**KY419204**	08-102-701626/2016/GII.2	2016
**KY419203**	08-102-518496/2016/GII.2	2016
**KY419202**	08-102-500116/2016/GII.2	2016
**NC039476**	Env/CHN/2016/GII.P16-GII.2/BJSMQ	2016
**LC325822**	Hu/GII/JP/2016/GII.P16-GII.2/AichiF194	2016
**LC325821**	Hu/GII/JP/2016/GII.P16-GII.2/AichiF168	2016
**MT126387**	109915/ATH/GII.2	2016
**MT126389**	98562/ATH/GII.2	2016
**MT126393**	108094/ATH/GII.2	2016
**MT126408**	110087/ATH/GII.2	2016
**MT126396**	44230/ATH/GII.2	2016
**LC213899**	Hu/GII/JP/2016/GII.P16-GII.2/Ibaraki607	2016
**MT126380**	62032/ATH/GII.2	2017
**MF775369**	Hu/GII.2/Tainan/16-7/2017/TW	2017
**MF775368**	Hu/GII.2/Miaoli/21-11/2017/TW	2017
**KY596003**	Hu/GII.2/Pingtung/10-28/2017/TW	2017
**LC325833**	Hu/GII/JP/2017/GII.P16-GII.2/AichiF15	2017
**LC325832**	Hu/GII/JP/2017/GII.P16-GII.2/AichiF318	2017
**LC325831**	Hu/GII/JP/2017/GII.P16-GII.2/AichiF305	2017
**LC521542**	2018/TH/S8_110	2018
**LC521530**	2018/TH/S4_81-1	2018
**LC521527**	2018/TH/S3_48	2018
**MH279486**	Hu/GII.P16_GII.2/Chiayi/20-8/2018/TW	2018
**LC521596**	2019/TH/S14_113	2019
**LC521593**	2019/TH/S16_112	2019
**MK571446**	2019-2-20-23/2019/GII.P16_GII.2	2019
**LC646333**	Hu/GII.2[P16]/1047F/Tokyo/2020/Japan	2020
**LC646332**	Hu/GII.2[P16]/1192F/Tokyo/2020/Japan	2020
**LC646331**	Hu/GII.2[P16]/1185/Tokyo/2020/Japan	2020
**LC644982**	Hu/GII.2[P16]/1262/Tokyo/2021/Japan	2021
**LC644981**	Hu/GII.2[P16]/1237/Tokyo/2021/Japan	2021
**LC644979**	Hu/GII.2[P16]/1453/Tokyo/2021/Japan	2021

Temporal signal evaluationby the root-to-tip analysis results, using TempEst program in Beast v1.10.4 package is shown at the following figure:

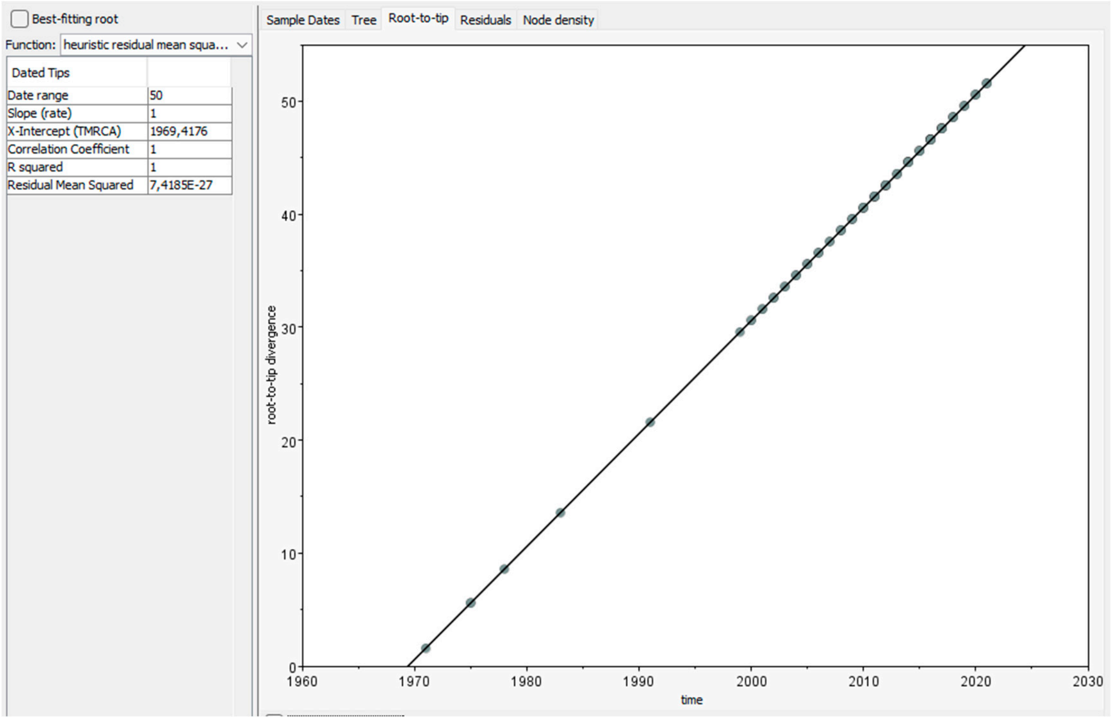

Regarding the parameters used for MCC tree construction, model selection and temporal tree construction using the Bayesian method, the procedure described below was followed:

First of all, a nexus format alignment of the GII.4 strains was produced using the MEGA XI software. The appropriate file was then imported into the BEAUti software, implemented in the BEAST v1.10.4 software package. Following parsing of date values for all strains, the evolutionary model was set using the HKY substitution model, with estimated base frequencies, the Gamma Site Heterogeneity model with 4 Gamma categories and 3 partitions into codon positions 1, 2, 3. The parameters of unlink substitution rate parameters across codon positions and unlink rate heterogeneity model across codon positions were selected. A strict clock type was selected, with Coalescent: Constant size tree prior and Random starting tree. The prior settings were as follows:

Parameter	Prior	Bound	Description
CP1.kappa	* LogNormal [1, 1.25], initial=2	[0, ∞]	HKY transition-transversion parameter for codon position 1
CP2.kappa	* LogNormal [1, 1.25], initial=2	[0, ∞]	HKY transition-transversion parameter for codon position 2
CP3.kappa	* LogNormal [1, 1.25], initial=2	[0, ∞]	HKY transition-transversion parameter for codon position 3
frequencies	* Dirichlet [1,1]	[0, ∞]	basefrequencies
CP1.alpha	* Exponential [0.5], initial=0.5	[0, ∞]	gamma shape parameter for codon position 1
CP2.alpha	* Exponential [0.5], initial=0.5	[0, ∞]	gamma shape parameter for codon position 2
CP3.alpha	* Exponential [0.5], initial=0.5	[0, ∞]	gamma shape parameter for codon position 3
allNus	* Dirichlet [1,1]	[0, ∞]	relative rates amongst partitions parameter
clock.rate	* Approx. ReferencePrior, initial=1	[0, ∞]	substitutionrate
treeModel.rootHeight	* UsingTreePrior in [45, ∞]	[45, ∞]	root height of the tree
constant.popSize	* 1/x, initial=1	[0, ∞]	coalescentpopulationsizeparameter

The operators of the parameters were as follows:

Operates on	Type	Tuning	Weight	Description
CP1.kappa	scale	0.75	1.0	HKY transition-transversion parameter for codon position 1
CP2.kappa	scale	0.75	1.0	HKY transition-transversion parameter for codon position 2
CP3.kappa	scale	0.75	1.0	HKY transition-transversion parameter for codon position 3
frequencies	deltaExchange	0.01	1.0	frequencies
CP1.alpha	scale	0.75	1.0	gamma shape parameter for codon position 1
CP2.alpha	scale	0.75	1.0	gamma shape parameter for codon position 2
CP3.alpha	scale	0.75	1.0	gamma shape parameter for codon position 3
clock.rate	scale	0.75	3.0	substitutionrate
Substitutionrate and heights	upDown	0.75	3.0	Scales substitution rates inversely to node heights of the tree
allNus	deltaExchange	0.01	3.0	Change partition rates relative to each other maintaining mean
Tree	subtreeSlide	1.0	30.0	Performs the subtree-slide rearrangement of the tree
Tree	narrowExchange	n/a	30.0	Performs local rearrangements of the tree
Tree	wideExchange	n/a	3.0	Performs global rearrangements of the tree
Tree	wilsonBalding	n/a	3.0	Performs the Wilson-Balding rearrangement of the tree
treeModel.rootHeight	scale	0.75	3.0	root height of the tree
Internal nodeheights	uniform	n/a	30.0	Draws new internal node heights uniformally
Tree	subtreeLeap	1.0	103.0	Performs the subtree-leap rearrangement of the tree
Tree	subtreeJump	1.0	10.3	Performs the subtree-jump rearrangement of the tree
constant.popSize	scale	0.75	3.0	coalescentpopulationsizeparameter

The MCMC options were according to the following figure:

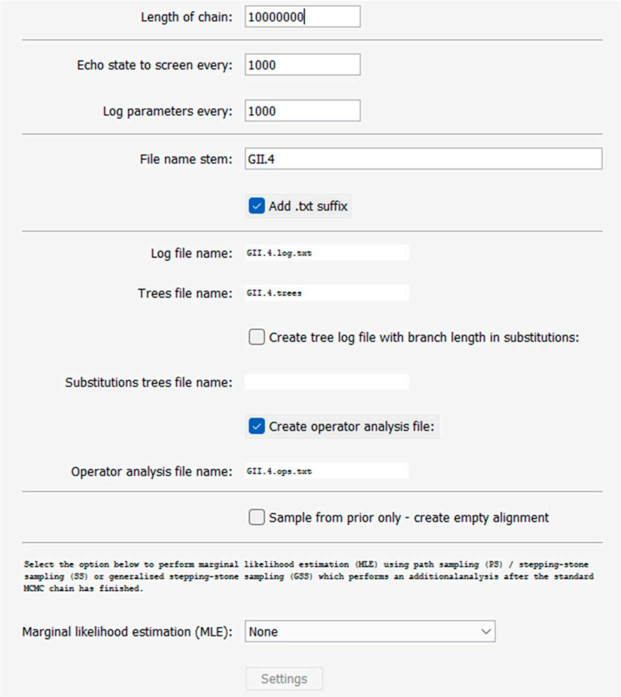

The BEAST .xml file that was produced was uploaded to the BEAST software. The generated .tree files were annotated with the software TreeAnnotator, included in the BEAST package, with burnin as the number of trees set to 200, posterior probability limit to 0.0 and the maximum clade credibility option selected. The generated tree nexus file format was finally uploaded to the FigTree v1.4.4 software for the production of the temporal phylogenetic analysis.
